# *Malassezia* and Parkinson's Disease

**DOI:** 10.3389/fneur.2019.00758

**Published:** 2019-07-24

**Authors:** Martin Laurence, Julián Benito-León, Frédéric Calon

**Affiliations:** ^1^Shipshaw Labs, Montreal, QC, Canada; ^2^Department of Neurology, University Hospital “12 de Octubre”, Madrid, Spain; ^3^Department of Medicine, Faculty of Medicine, Complutense University, Madrid, Spain; ^4^Centro de Investigación Biomédica en Red Sobre Enfermedades Neurodegenerativas, Madrid, Spain; ^5^Faculty of Pharmacy, Université Laval, Quebec City, QC, Canada; ^6^Neurosciences Unit, CHU de Québec-Université Laval Research Center, Quebec City, QC, Canada

**Keywords:** Parkinson's disease, seborrheic dermatitis, *Malassezia*, immunodeficiency, immunosenescence

## Abstract

Parkinson's disease (PD) is a common debilitating neurodegenerative disease caused by a loss of dopamine neurons in the substantia nigra within the central nervous system (CNS). The process leading to this neuronal loss is poorly understood. Seborrheic dermatitis (SD) is a common benign inflammatory condition of the skin which mainly affects lipid-rich regions of the head and trunk. SD is caused by over proliferation of the lipophilic fungus *Malassezia*. PD and SD are strongly associated. The increased PD risk following an SD diagnosis (OR = 1.69, 95% CI 1.36, 2.1; *p* < 0.001) reported by Tanner and colleagues remains unexplained. *Malassezia* were historically considered commensals confined to the skin. However, many recent studies report finding *Malassezia* in internal organs, including the CNS. This raises the possibility that *Malassezia* might be directly contributing to PD. Several lines of evidence support this hypothesis. AIDS is causally associated with both parkinsonism and SD, suggesting that weak T cell-mediated control of commensal microbes such as *Malassezia* might contribute to both. Genetic polymorphisms associated with PD (*LRRK2, GBA, PINK1, SPG11, SNCA*) increase availability of lipids within human cells, providing a suitable environment for *Malassezia*. Four *LRRK2* polymorphisms which increase PD risk also increase Crohn's disease risk; Crohn's disease is strongly associated with an immune response against fungi, particularly *Malassezia*. Finally, *Malassezia* hypha formation and melanin synthesis are stimulated by L-DOPA, which could promote *Malassezia* invasiveness of dopamine neurons, and contribute to the accumulation of melanin in these neurons. Although *Malassezia*'s presence in the substantia nigra remains to be confirmed, if *Malassezia* play a role in PD etiology, antifungal drugs should be tested as a possible therapeutic intervention.

## Introduction

Worldwide, about 20 out of 100,000 people are diagnosed with Parkinson's disease (PD) every year (1, 2). Prevalence in persons over 65 years of age is about 1.8% (3), and overall prevalence is expected to rise with aging demographics (4). Loss of dopamine in PD was discovered 60 years ago (5), and still provides the basis of therapy with its precursor, L-DOPA (6). However, the etiology of PD remains elusive, hindering the development of additional disease modifying treatments. Only 3–5% of PD cases are explained by known genetic variants (7), indicating that the majority of cases are sporadic and are likely influenced by environmental factors (7). An infectious component in PD has been suspected since the 1920s, because parkinsonism often followed encephalitis lethargica (8). The potential role of microbes and viruses in neurological diseases is becoming increasingly recognized, including in multiple sclerosis (9), Alzheimer's disease (10) and PD (11, 12). Some research groups specifically suspect fungal infections might be involved in central nervous system (CNS) diseases including Alzheimer's disease (13–15), amyotrophic lateral sclerosis (13, 16, 17), multiple sclerosis (18, 19), and schizophrenia (20–22).

Microbes and viruses are present in all humans from birth. While enteric bacteria have received the most attention from the research community, histological and molecular evidence of bacteria, fungi, and viruses have been found in organs previously considered mostly sterile, including the CNS (14–16, 23–25). Although most microbes are usually benign, some can become pathogenic under certain conditions. Propitious conditions allowing commensals to become pathogens are varied in nature, and include an overabundance of nutrients required for microbial growth, as well as immunodeficiency. Though immunodeficiency has been most widely studied in human immunodeficiency virus (HIV) patients (26–32), genetic or congenital immunological defects (33–37), the use of immunomodulatory drugs (38–40), and age-related immunosenescence (40, 41) all increase the risk of disease caused by normally well-tolerated microbes.

When both an overabundance of nutrients required for microbial growth and immunodeficiency occur in the same individual, this greatly favors microbial over proliferation. For example, the commensal lipophilic fungus *Malassezia furfur* can invade internal organs of immunodeficient infants who undergo lipid-rich parenteral nutrition, including the CNS (34). Seborrheic dermatitis (SD), a benign dermatological condition caused by over proliferation of *Malassezia* on the skin (29, 42, 43), is strongly associated with PD (44–46). Recent studies have found *Malassezia* DNA in the CNS of multiple sclerosis (MS) (23) and Alzheimer's disease patients (24). Could over proliferation of *Malassezia* in the CNS contribute to PD, as it does in SD and in immunodeficient infants undergoing lipid-rich parenteral nutrition? In this review, we explore this hypothesis in detail.

## *Malassezia* Primer

Despite being the most prevalent fungal genera in the human microbiome, *Malassezia* are little known outside the field of dermatology. *Malassezia* were first described in 1889, yet it took nearly a century to firmly establish their role in dandruff and seborrheic dermatitis (47). This delay can be attributed to *Malassezia*'s difficulty growing in culture (they require just the right concentration of certain lipids) (48), their extremely high prevalence on the skin (~100% throughout life) (49), and their resistance to fungicides (commonly used antifungal drugs do not completely eradicate *Malassezia* and their populations rebound once such treatments are discontinued) (50). About 50% of adults have first-hand experience with *Malassezia*-associated symptoms: they use antifungal shampoo to keep their dandruff under control, and whenever they switch back to normal shampoo, *Malassezia* and dandruff return (47).

Until very recently, it was believed that *Malassezia* were restricted to the skin, except in rare immunocompromised or lipid-rich parenteral nutrition cases (34, 51). Due to improved microbe detection techniques, many groups now report finding *Malassezia* within the body of both healthy adults and immunocompetent patients with various ailments (14–16, 23–25, 33, 52–61). *Malassezia*'s potential role in diseases of internal organs is just coming to light (19, 54, 62–64). It is important to note that *Malassezia*'s presence is not synonymous with disease: in the vast majority of individuals, *Malassezia* colonize the body without causing symptoms. This means detecting *Malassezia* in a given organ is far from sufficient to prove their involvement in diseases of that organ. Additional lines of evidence are necessary to implicate them, for example the efficacy of antifungal drugs in treating the disease, immunological evidence (such as antibodies against *Malassezia*) and genetic evidence (such as genes affecting *Malassezia*'s lipid supply or the immune response against fungi).

Since *Malassezia* are not well-known in the field of neurodegenerative disorders, this section is included as a primer on *Malassezia*'s suspected role in SD, acquired immune deficiency syndrome (AIDS), Crohn's disease (CD), spondyloarthritides (SpA), and MS. Each of these conditions is informative for PD.

### *Malassezia* Are a Necessary Factor in Seborrheic Dermatitis

*Malassezia*'s role in SD is now generally accepted (29, 42, 43, 50). Given the right conditions, *Malassezia* over proliferate on the skin (65), resulting in SD—though specific mechanisms are still open to debate (29, 42, 43, 50). Most SD cases respond well to topical fungicides which reduce *Malassezia* populations on affected patches of skin to levels tolerated by patients (29, 42, 43).

SD occurs mainly in lipid-rich skin regions, especially the face, trunk and scalp (29, 42, 43). *Malassezia* are lipid-dependent fungi: they lack key lipid metabolism genes (including fatty acid synthase, stearoyl-CoA desaturase, and enoyl-CoA isomerase), and thus depend on host lipids for survival (48). Skin lipid production varies during our lifetime, with a peak in the first year of life, followed by a second peak in adolescence (66–68): production is depressed during the rest of childhood, which corresponds to the period of lowest SD risk (69). In adults, the risk of SD increases substantially with age (69). This is unexpected because skin lipid levels slowly decline with age (67), so *Malassezia* should have increasing difficulty securing lipids in the elderly.

Azathioprine and cyclosporin, two immunosuppresive drugs which target T cells, substantially increase SD risk (39). Similarly, CD4+ T cell counts are inversely associated with SD risk and severity in AIDS patients (29). Peripheral blood mononuclear cells (PBMC) from SD patients produce less IL-2 and interferon gamma (IFNγ) when exposed to *Malassezia* antigens as compared to age-matched controls (70), suggesting a weak type 1 helper T cell (Th1) response against *Malassezia* is a characteristic of SD. In healthy individuals, thymic involution reduces naive T cell production, which results in a slow decline in T cell efficacy over our lifespan (71). This can be observed in part by measuring T cell receptor (TCR) diversity (71, 72). T cell immunosenescence can explain why SD risk increases with age, despite declining skin lipids. SD seems to be mainly due to the combination of ample lipids (29, 42, 43) and weak T cell-mediated control (70, 73, 74) of *Malassezia*, which together allow this fungus to over proliferate on the skin.

Seborrheic dermatitis (SD) is a well-known symptom associated with PD: PD patients have ~50% SD prevalence, while controls only have ~3% prevalence (44, 45). Though these are the most frequently cited figures, they are based on old studies whose accuracy has drawn criticism (75). We only found one recent study which measured the association between PD and SD (46). It reported that SD increases the risk of a *subsequent* PD diagnosis (OR = 1.69, 95% CI 1.36, 2.1; *p* < 0.001). This association remained significant when the SD diagnosis was made at least 5 years before the PD diagnosis, suggesting PD-associated treatments or behavior cannot explain it (46). This study reported an SD diagnosis rate of 4% prior to a PD diagnosis, as compared to 2.5% for age matched controls (46). This low rate of association indicates factors predisposing to SD and PD are mostly different. Of note, this report (46) is only available as an abstract and has not been published as a peer-reviewed article. Specific mechanisms underlying the association between SD and PD are not known (75). Nonetheless, this association suggests that mechanisms allowing *Malassezia* to over proliferate and cause SD (ample lipids and T cell immunosenescence) should be thoroughly investigated as possible mechanisms underlying PD as well.

### *Malassezia* Over Proliferate in AIDS

Over proliferation of normally well-tolerated microbes is frequently observed in immunodeficient patients. The very first symptoms of AIDS often involve the over proliferation of fungi, such as *Candida* in the mouth (27), *Malassezia* on the skin (28, 29), or *Pneumocystis* in the lungs (30). Such fungi are normally present in healthy individuals (49, 76, 77), and are considered benign members of the human microbiome.

Latent microbes which are not considered part of the normal microbiome can also cause severe illness in AIDS patients. *Toxoplasma gondii* is a protist which colonizes the CNS of a large subset of the population for life (31). It rarely causes severe symptoms in immunocompetent individuals, but often causes life-threatening encephalitis in AIDS patients or during immunosuppression (31). Though much rarer today, *Treponema pallidum* (syphilis) also colonizes the CNS of infected individuals for life, typically without causing symptoms until old age (78). AIDS hastens neurosyphilis in previously asymptomatic *Treponema pallidum* carriers, placing young patients at an unusually high risk of this disease (32).

These well-studied secondary infections in AIDS indicate that CD4+ T cell-mediated control of latent microbes—including *Toxoplasma gondii* and *Treponema pallidum* in the CNS—is critical for our health. *Malassezia* have only recently been recognized as present within the body, so the effect of AIDS on *Malassezia* populations beyond the skin has not been investigated. The only such study we could find reported marked increase in *Malassezia* levels in stool of AIDS patients (51). This suggests CD4+ T cell-mediated control of *Malassezia* may be necessary to maintain well-tolerated *Malassezia* populations within the body, as well as on the skin. If *Malassezia* are involved in diseases of internal organs, AIDS would be expected to precipitate such diseases in genetically susceptible individuals—similarly to how it precipitates SD by allowing *Malassezia* to over proliferate on the skin. Interestingly, recent studies (reviewed in the next two sections) implicate *Malassezia* in arthritis (62) and demyelination (19), two common AIDS symptoms where no secondary infections were thought to be present.

AIDS increases the risk of developing early onset parkinsonism (79–82). Unlike sporadic PD, AIDS-associated parkinsonism progresses rapidly and affects young individuals (79). This raises the possibility that if *Malassezia* are present in the CNS (23, 24), their over proliferation might contribute to parkinsonism in AIDS patients, in a similar way that *Malassezia* on the skin contribute to SD in these patients (28, 29). This putative mechanism implies that poor CD4+ T cell-mediated control of *Malassezia* populations would be an important precipitating factor for PD and SD, occurring gradually in normal aging (through immunosenescence) and much more suddenly in AIDS (through lack of CD4+ T cells).

### *Malassezia* Are a Necessary Factor in Crohn's Disease and Spondyloarthritides

Spondyloarthritides (SpA) are a group chronic immune-mediated diseases mainly driven by alpha beta T cells recognizing intracellular peptides through HLA-B^*^27 presentation (62, 83). Affected organs include the spine, joints, skin, eyes, gut, and prostate (62). Historically, isolated inflammation of the eyes, gut, and skin—respectively acute anterior uveitis, inflammatory bowel disease (Crohn's disease [CD] and ulcerative colitis) and psoriasis—were considered separate diseases unrelated to SpA. However, SpA, acute anterior uveitis, inflammatory bowel disease and psoriasis run together in families (84), and share many polymorphisms in genes controlling T cell activation (85), strongly suggesting that they are the same immunological pathology (84). In particular, the fact that HLA-B^*^27 increases the risk of each disease strongly suggests the same antigens are being targeted (62). Varied lines of evidence support the presence of an elusive necessary intracellular fungal infection in each affected organ, which is efficiently detected by HLA-B^*^27 and CARD9 (62). CARD9 is an essential signaling protein for fungal immunity: homozygous loss-of-function CARD9 mutations cause severe mycoses (37). CARD9 polymorphisms are associated with inflammatory bowel disease and SpA (62, 64). Oral antifungal drugs are effective in psoriasis (86–89), psoriatic arthritis (89, 90), and likely in Crohn's disease as well (91).

In inflammatory bowel disease and psoriasis, strong evidence points to *Malassezia* as being the causative genus (62). Enteric *Malassezia* is strongly associated with CD (54, 64) and ulcerative colitis (63). Immune recognition of *Malassezia* occurs specifically through CARD9 in the gut, and knocking out CARD9 in mice abrogates colitis symptoms following exposure to *Malassezia* (64). In CD, CARD9 risk alleles increase PBMC secretion of tumor necrosis factor alpha (TNF-α) following *Malassezia* antigen challenges (64). Antibodies against *Malassezia* are associated with CD (64) and psoriasis (92, 93). Applying lysed *Malassezia* to the skin provokes psoriasis lesions in susceptible individuals (94). The only known fungus which is commonly present in the gut (61) and on the skin (49), and thus can explain why Crohn's patients develop psoriasis during vedolizumab treatment (95), is *Malassezia*. Finally, PBMCs in psoriasis react strongly to *Malassezia* antigens (96). These data suggest that a dysregulated immune response against *Malassezia* in the gut is causative in CD (64).

CD and SpA provide three key insights for PD. First, they demonstrate that *Malassezia* can cause diseases of internal organs in genetically predisposed individuals. Second, the increased risk of psoriasis in overweight individuals (97–100), and the increased risk of CD in carriers of certain *LRRK2* alleles (101, 102) are most simply explained by enhanced lipid availability (103) which promotes *Malassezia's* growth by supplying it with lipids [the same *LRRK2* alleles increase PD risk (101, 102)]. Third, intracellular melanin reminiscent of neuromelanin (104) is associated with inflammation of the prostate (104–106). Though indigenous production of melanin by human cells has been proposed as an explanation (104), a second possible origin would be from *Malassezia* which have colonized the prostate and CNS. *Malassezia* produce DOPA-melanin from L-DOPA (107). Both prostate epithelial cells and dopamine neurons contain intracellular lipid droplets which can fulfill *Malassezia*'s requirement for lipids.

### *Malassezia* Are Found in the CNS in Multiple Sclerosis

MS has many direct links with the immune response against fungi (18) and with SpA (62). The distribution of the age at onset of MS (108) is nearly identical to ankylosing spondylitis (109) and CD (110). The fungicidal compound dimethyl fumarate is effective in psoriasis (111), psoriatic arthritis (89, 90), and MS (112). MS is moderately associated with SpA (113, 114), inflammatory bowel disease (115, 116), and psoriasis (115, 117). These associations are surprising because SpA shares few genetic susceptibility loci with MS (85), and unlike SpA, MS is mainly B cell-mediated (18). The simplest explanation is that MS shares a *necessary* environmental factor with SpA (113), such as colonization of internal organs by *Malassezia* (19).

A recently published study compared fungi in the CNS of MS patients vs. controls, and found *Malassezia* in 9 of 10 MS cases, and in 1 of 9 controls (OR = 72, 95% CI 3.8–1,350, *p* = 0.0011) (23). Myelin producing Schwann cells are lipid-rich (118), thus can fulfill *Malassezia*'s requirement for lipids.

Unlike in SpA, direct links between *Malassezia* and MS are currently limited to a single study (23). The role of *Malassezia* in MS is mainly supported by the many associations between MS and SpA (62), which suggest the same fungal infection is necessary for both (62). This means *Malassezia* likely cross the blood-brain-barrier and colonize the CNS. The closely related fungus *Cryptococcus neoformans* survives phagocytosis, and uses macrophages to move within the body and CNS (119). A recent study reported that *Malassezia* also survive phagocytosis (120), suggesting they might use macrophages to reach the CNS.

## Seborrhea and Parkinson's Disease

Seborrhea is defined as an elevated sebum secretion rate (SER) (121). To explain the association between SD and PD, Burton and colleagues proposed that SER could be increased by neuroendocrine stimulation via the parasympathetic system (122). Seventeen years later, the same group stated that “There is no evidence of a neural control of sebaceous function,” abandoning this hypothesis (123). This hypothesis was also excluded by Martignoni and colleagues who found little evidence of increased SER in PD (124), nor any relation with symptom severity or treatment type (124): “Sebum excretion does not appear to be related to the abnormalities of the autonomic nervous system” (124).

The two largest studies of SER in PD found no association in women (124, 125). Both studies stratified male subjects and found an association in only a subset of men (124, 125). Smaller studies reported conflicting results (126–128). Some studies reported that the administration of levodopa decreased SER (122, 125, 129), while others found no effect (124, 130).

Seborrhea seems to have little to do with SD because SER is unrelated to SD (121, 123)—although seborrhea and SD occur in the same skin regions, increased lipid secretion rates are not a characteristic of SD, strongly suggesting that seborrhea and SD are completely different clinical entities (121, 123). *Malassezia* can cause SD under a wide range of sebum conditions (123), and a reduction in sebum level below the physiological range is required to abrogate SD symptoms (123). This lead Cowley and colleagues to propose that limited movement might be one of the main causes of SD observed in a variety of neurological disorders, including PD (123). This hypothesis is strongly supported by SD onset observed shortly after cervical cord injuries (131), which was attributed to changes in hygiene rather than changes in SER (131).

Though the link between subjective seborrhea and PD has been reported in older studies (75), the fact that seborrhea and SD were considered synonymous (75) means these studies cannot be used to evaluate SER in PD. Therefore, we have found no compelling evidence of increased SER in PD. If such an increase exists, it must be of a small magnitude, requiring large studies to detect, and measure.

Moreover, because SER is not associated with SD (121), small changes in SER are an unlikely explanation for the association between PD and SD (123). Changes in SER fail to explain the increase in SD risk with age (69): SER decreases 2-fold between puberty and 50 years of age (67). Weak T cell-mediated control of *Malassezia* thus appears to be the main contributing factor to SD in older adults (70, 73, 74). This means reduced movement is a much more plausible contributor to SD in neurological diseases (123) and spinal cord injuries (131) than increased SER. We propose two non-SER mechanisms which could explain how reduced movement increases SD risk: [1] changes in hygiene might promote *Malassezia*'s growth, possibly by allowing sebum to accumulate; [2] reduced movement hampers lymph flow (132), which might prevent CD4+ T cells controlling *Malassezia* populations from activating and reaching the skin. Regardless of the mechanism of SD in movement-impaired individuals, an SD diagnosis increases the risk of a PD diagnosis many years later (46), suggesting that reduced movement cannot entirely explain the association between PD and SD.

## *Malassezia* and Parkinson's Disease

Beyond the strong association between PD and SD, and preliminary evidence of *Malassezia*'s presence inside the CNS, three additional lines of evidence support a direct contribution of *Malassezia* in PD: [1] many PD risk alleles affect lipid metabolism (*Malassezia* are lipophilic), [2] *Malassezia* invasiveness and melanin production are both stimulated by L-DOPA (L-DOPA is naturally abundant in the substantia nigra), and [3] low CD4+ T cell counts observed in PD might contribute to the over proliferation of microbes such as *Malassezia*.

### PD Genetics and Lipids

*Malassezia* are lipid-dependent fungi which consume host lipids by secreting extracellular lipases (48, 50, 133). On the skin, they consume extracellular lipids in sebum (50), though they can also utilize intracellular lipids by invading keratinocytes (133). The best documented cases of *Malassezia* inside the body were reported following lipid-rich parenteral nutrition (34). In these cases, *Malassezia* were mainly found within the wall of arteries which contained lipid deposits, suggesting high lipid concentrations are required to sustain *Malassezia* in tissue (134).

If *Malassezia* reach the CNS, ample access to lipids might allow them to take-hold or over proliferate, as observed in arteries during lipid-rich parenteral nutrition and on the skin in SD. Among other cellular functions, genes associated with PD risk often impact lipid metabolism. Polymorphisms in *GBA, LRRK2*, and *PINK1*—three important genetic risk factors of PD (135)—increase the concentration of intracellular lipids. *GBA* risk alleles have been most studied in Gaucher's disease, an autosomal recessive disorder which is caused by an abnormal accumulation of lipids in lysosomes due to defects in the β-glucocerebrosidase enzyme (which *GBA* encodes) (136). β-glucocerebrosidase hydrolyses the beta-glucosidic linkage of glucocerebroside, breaking it into ceramide and glucose (136). This is a key step in lipid metabolism: glucocerebroside accumulates within cells—especially macrophages—in the absence of β-glucocerebrosidase (136). Glucocerebroside typically contain long chain saturated fatty acids (137). *Malassezia* lack fatty acid synthase genes, so they depend on an external source of long-chain saturated fatty acids to grow (48, 138). We speculate that cells which contain high amounts of glucocerebroside are more susceptible to colonization by *Malassezia*. *LRRK2* risk allele Y1699C phosphorylates Rab8a at T72, which promotes formation of large lipid droplets (103). *PINK1* risk alleles increase the size and count of lipid droplets by reducing PGC-1a-mediated mitochondrial fatty acid oxidation (139). The newly recognized genetic risk factor *SPG11* also causes an abnormal accumulation of lipids in lysosomes (140). *SNCA* risk alleles, another important genetic risk factor of PD, render lipid droplets more permeable to lipases (141) The alpha-synuclein variants they encode are less effective at coating such droplets (141), perhaps giving *Malassezia* easier access to this essential nutrient source, which they consume by secreting lipases (48, 50, 133).

Interestingly, four *LRRK2* risk alleles in PD also increase the risk of CD (101, 102). CD is likely caused by a T cell-mediated immune response against *Malassezia* in the gut (62, 64). It is important to note that only *LRRK2* PD risk allele Y1699C has been tested for lipid-droplet formation, and that *LRRK2* alleles associated with both CD and PD (N551K, R1398H, N2081D, G2019S) have not been tested for excess lipid-droplet formation. It is also important to note that the most important *LRRK2* PD risk allele G2019S did not reach statistical significance in one of the CD studies (101): “The LRRK2 N2081D CD risk allele is located in the same kinase domain as G2019S, a mutation that is the major genetic cause of familial and sporadic PD […]. Notably, G2019S did not have nominally significant CD association (*P* = 0.12), likely because of subtle stochastic fluctuations in allele frequencies during imputation.” However, it amply reached statistical significance (*p* = 0.0014) in the other CD study (102).

### L-DOPA and Melanin

Neuromelanin is abundantly present in neurons of the substantia nigra (142, 143). Though neuromelanin's precise role in PD pathogenicity is not known, neurons containing this pigment seem to die as the disease progresses (144, 145).

Neuromelanin concentration and distribution within dopamine neurons increase with age, beginning in childhood (142, 143). The origin and biochemical process through which neuromelanin is synthesized remains a matter of debate, though the simplest explanations of tyrosinase-mediated synthesis and autoxidation have been deemed unlikely (144). In contrast with peripheral melanin, neuromelanin is often collocated with lipids [in particular dolichols (146)] and is not produced by nor kept within melanosomes (144).

Intriguingly, *Malassezia* was found *in vitro* to produce melanin from exogenous L-DOPA (107). In addition, L-DOPA triggered hyphal growth (107) which is thought to be the most invasive *Malassezia* morphology, allowing it to penetrate host cells and tissue. This raises the possibility that *Malassezia*, if present in the CNS, would be more invasive in naturally L-DOPA rich regions such as catecholaminergic neurons of the substantia nigra (147). Neuromelanin in the substantia nigra might be produced by *Malassezia* following invasion of dopamine neurons, as *Malassezia* consume intracellular lipid droplets in these neurons. Since neurons are long-lived, melanin of fungal origin could accumulate following repeated transient exposure to *Malassezia*. A similar mechanism could explain the presence of melanin in the basal region of prostate secretory epithelial cells, where lipid droplets are found (104).

### AIDS and CD4+ T Cells

AIDS patients are at increased risk of parkinsonism (79): about 5–15% are affected (82). AIDS-induced parkinsonism differs from sporadic PD in several respects: bradykinesia and rigidity are usually symmetrical, postural instability and gait abnormalities occur earlier, and onset can occur at a much younger age (79).

A small post-mortem study reported a higher fraction of pigmented cells in the pars compacta of the substantia nigra in AIDS patients (as compared to age-matched controls) (80). It also reported lower neuronal density in AIDS patients, suggesting neuronal loss (80). Another small post-mortem study reported lower dopamine levels in the caudate nucleus tissue in AIDS patients (as compared to age-matched controls) (81), which was attributed to the loss of nigrostriatal dopamine neurons.

AIDS-induced parkinsonism usually occurs when CD4+ T cell counts are very low (82). Interestingly, peripheral blood CD4+ T cell counts in sporadic PD are also depressed (148–150), though not as much as in AIDS. This suggests CD4+ T cells are protective in PD, and prevent or slow dopamine neuron loss. Age-related CD4+ T cell immunosenescence could thus partly explain why PD risk increases with age.

**Figure 1:**
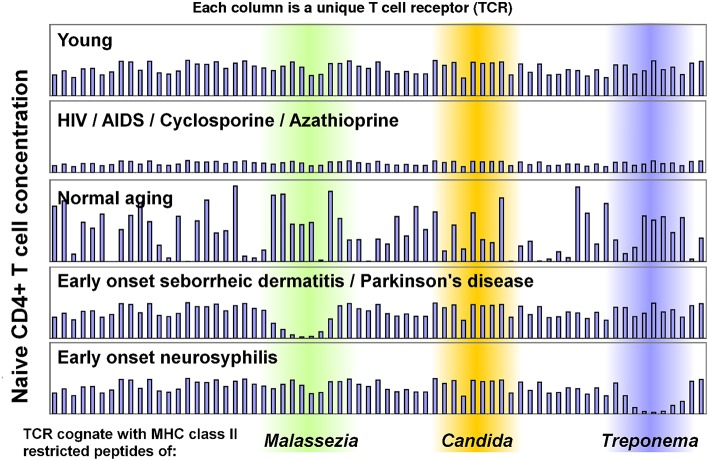
Model of CD4+ T cell immunosenescence, which illustrates how CD4+ T sensitivity to different microbes can change. Each column represents a sub-population of naïve CD4+ T cells which has an identical T cell receptor (TCR). Some TCRs are cognate with *Malassezia* peptides (green), others with *Candida* peptides (orange) or *Treponema* peptides (blue). Recognized peptides will vary between individuals based on their MHC Class II alleles. This model can explain early onset seborrheic dermatitis, parkinsonism and neurosyphilis in AIDS patients, as the concentration of *all* TCRs decline. It can also explain why seborrheic dermatitis and Parkinson's disease are so strongly associated in immunocompetent individuals: the concentrations of TCRs cognate with *Malassezia* peptides decline, allowing *Malassezia* to over proliferate due to a weak CD4+ T cell-mediated immune response. This has been measured in seborrheic dermatitis (70), but not in Parkinson's disease. These data were manually generated for illustrative purposes only (they are not measurements from patients).

While most human cells are genetically identical, CD4+ T cells have different TCR genes produced through V(D)J recombination, allowing subsets of CD4+ T cells to recognize specific MHC class II restricted peptides. This means a small subset of CD4+ T cells recognize *Malassezia* peptides, another small subset recognizes *Candida* peptides, and so on (Figure 1). In AIDS, CD4+ T cell subsets recognizing each microbial species are equally vulnerable to depletion (Figure 1). This results in accrued susceptibility to a variety of infections which would not usually be life-threatening, but can prove fatal when they affect a vital organ of an AIDS patient.

**Figure 2:**
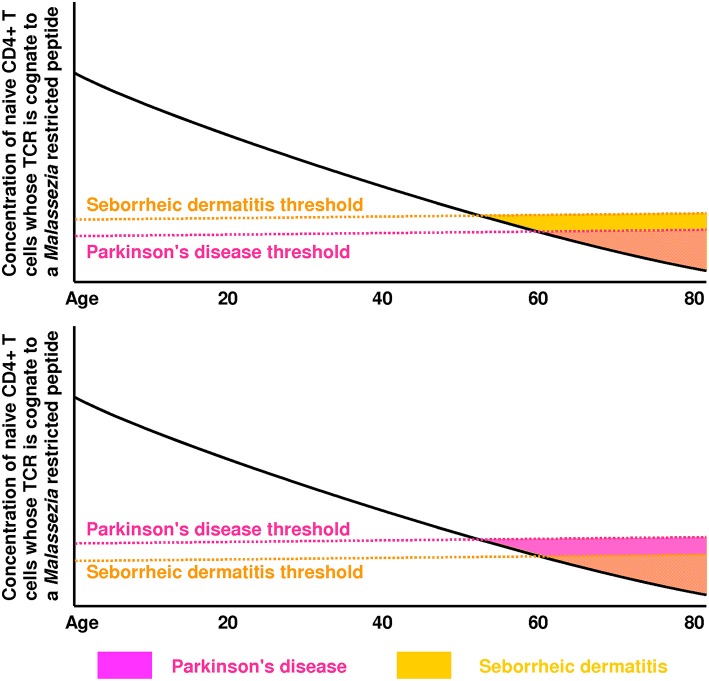
These plots illustrate how the green region of Figure 1 is expected to vary with age (mainly due to thymic involution and immunosenescence). In the top plot, the seborrheic dermatitis (SD) threshold is reached first, which means SD will precede Parkinson's disease (PD). In the bottom plot, the PD threshold is reached first, which means PD will precede SD. These thresholds are likely influenced by genes controlling the strength of Th1 immunity against *Malassezia*. The PD threshold could also be affected by lipid levels (modulated by *GBA, LRRK2, PINK1, SPG11*) and lipid droplet accessibility by lipases (modulated by *SNCA*). The low rate of SD diagnosis preceding a PD diagnosis [4%] reported by Tanner and colleagues (46) suggests that PD and SD share few genetic/environmental risk factors, and that the PD threshold is usually crossed first in individuals who develop PD.

Though low CD4+ T cell counts in PD (148–150) suggest that the concentration of *all* CD4+ T cells matters, we propose that the concentration and efficacy of the CD4+ T cell subset which recognizes *Malassezia* peptides is the most important precipitating factor in PD and SD (Figure 2). This provides a simple explanation for the marked increase in risk of both PD and SD in AIDS patients, and for the strong epidemiological association between PD and SD in immunocompetent individuals.

## Conclusion

The strong epidemiological association between PD and SD suggests these two diseases share risk factors or underlying mechanisms (Table 1). The best established shared risk factor is AIDS, which greatly increases the risk of both parkinsonism and SD in young individuals. A weak CD4+ T cell-mediated immune response thus seems to be a key common mechanism. SD occurs mainly in lipid-rich areas of the skin (42), and several PD risk alleles either cause an abnormal accumulation of intracellular lipids (*GBA, LRRK2, PINK1, SPG11*) (103, 136, 139, 140) or increase lipase permeability of lipid droplets (*SNCA*) (141). This suggests a second shared mechanism is increased lipid availability. The main cause of SD is the over proliferation of the fungus *Malassezia* on the skin, which seems partly driven by a weak T cell-mediated immune response, and partly driven by an abundance of lipids which are required for *Malassezia*'s growth. The main cause or causes of PD are not known.

**Table 1:** Similarities between seborrheic dermatitis (SD) and Parkinson's disease (PD) risk factors.

	**Seborrheic dermatitis (SD)**	**Parkinson's disease (PD)**
High lipid levels (*Malassezia* are lipophilic fungi)	SD risk follows sebum levels in childhood (29, 42, 43) Sebum-rich skin areas are affected in SD (29, 42, 43)	*GBA* (151), *LRRK2* (103), *PINK1* (139), *SPG11* (140) PD risk alleles increase intracellular lipids *SNCA* PD risk alleles fail to protect lipid droplets from lipases (141)
Weak CD4+ T cell control of microbes (immunocompromised or immunosenescent)	AIDS is a major risk factor of SD (29, 73) Anti-T cell drugs (azathioprine and cyclosporin) are major risk factors of SD (39) Weak Th1 response against *Malassezia* measured in SD (70)	Low CD4+ T cell counts are associated with PD (148–150) AIDS is a major risk factor of parkinsonism in young individuals (79)
Direct involvement of *Malassezia*	Antifungals relieve SD symptoms (29, 42, 43) *Malassezia* are the only fungi commonly present on SD skin (29, 42, 43)	*Malassezia* reported present in CNS in other conditions (23, 24) (not tested in PD) L-DOPA stimulates *Malassezia* hypha formation and melanin production (107) Melanin of unknown origin present in dopamine neurons (144) and prostate epithelial cells (104) Fungal antigens are present in corpora amylacea in PD (13)

Evidence related to CD, SpA, and MS reviewed here support both the presence and potential pathogenicity of *Malassezia* in internal organs, including in the CNS. PD and CD share *LRRK2* susceptibility alleles (101, 102). Abnormal lipid droplet accumulation associated with the *LRRK2* Y1699C allele (103) suggests a genetically determined increased in intracellular lipids might enable *Malassezia* over proliferation in the CNS and gut. It is important to note however that abnormal lipid droplet accumulation has not been tested for any other *LRRK2* risk allele (103). Two very recent studies report finding *Malassezia* in the CNS in association with MS (23) and Alzheimer's disease (24). If *Malassezia* are found in the substantia nigra, this would strongly support the hypothesis that *Malassezia* are a necessary factor in PD.

Idiopathic AIDS symptoms such as parkinsonism, demyelination and arthritis could be explained by poor CD4+ T cell-mediated control of *Malassezia* populations in internal organs. Similarly, increased risk of PD and neurosyphilis in older adults could be explained by gradual immunosenescence, in particular the loss of CD4+ T cell subsets which control specific microbe populations such as *Malassezia* and *Treponema*. AIDS can be seen as a markedly accelerated form of immunosenescence, which precipitates certain diseases of aging in young individuals. The strong association between SD and PD, and the increased risk of SD and PD in AIDS patients, are most simply explained by waning CD4+ T cell control of *Malassezia* populations.

Using SD as a model disease (70), we do not expect to find strong immunological evidence of *Malassezia*'s role in PD, because demonstrating a *weak* immune response is more difficult than demonstrating a *robust* response. This contrasts with SpA and MS where even small studies report strong adaptive immune responses against fungi (18, 62). For example, interferon gamma (IFNγ) released by PBMC exposed to *Malassezia* is markedly elevated in psoriasis (96)—so much that this assay can be used as a reliable biomarker of psoriasis (96). IFNγ released by PBMC exposed to *Malassezia* is moderately depressed in SD (70)—this could also be used as biomarker of SD, though it would be much less sensitive and specific than in psoriasis. These biomarkers suggest psoriasis is partly caused by an exaggerated Th1 response against *Malassezia*, while SD is partly caused by an insufficient Th1 immune response against *Malassezia*—and the Th1 response against *Malassezia* in healthy skin is just right. We expect to find moderately depressed IFNγ release when PBMC from PD patients are exposed to *Malassezia*, while responses to other microbes are expected to be similar. We also expect this biomarker to be predictive of future PD risk.

Once *Malassezia*'s presence in the CNS is confirmed, and a weak Th1 response against *Malassezia* in PD patients who do not have SD is demonstrated, the key additional proof of *Malassezia*'s involvement will be the efficacy of CNS-penetrant antifungal drugs such as voriconazole in preventing PD or slowing PD progression. It is imperative to confirm the presence of *Malassezia* in the CNS, especially in the substantia nigra. Metagenomics is a promising new approach to detect microbes in clinical specimens (152–155), and could be applied to post-mortem CNS tissue to test for *Malassezia*. Several important idiopathic diseases have recently been attributed to previously unsuspected infections, such as *Helicobacter pylori* in gastric ulcers and human papillomaviruses in cervical cancer. In both cases, these discoveries led to effective new treatments. Confirming *Malassezia*'s role in PD would open many new treatment avenues.

## Author Contributions

First draft was written by ML after discussion with FC and JB-L. All the authors critiqued and revised the draft.

### Conflict of Interest Statement

The authors declare that the research was conducted in the absence of any commercial or financial relationships that could be construed as a potential conflict of interest.
